# Limitations of ATN-Based Diagnosis and the Need for an Integrated Clinical-Biological Staging Model

**DOI:** 10.1192/bjo.2026.11070

**Published:** 2026-06-30

**Authors:** Mahnoor Javed

**Affiliations:** Imperial College London, London, United Kingdom

## Abstract

**Aims:**

The Amyloid-Tau-Neurodegeneration (ATN) framework defines Alzheimer’s disease using primarily biological markers of pathology rather than clinical presentation. Although ATN classification has improved biological characterisation, new evidence indicates that biomarker stage is frequently discordant with clinical severity, functional impairment and prognosis, limiting its utility for personalised management planning. This poster aims to critically evaluate the strengths and limitations of ATN-based diagnosis and explore the role and management implications of an integrated clinical-biological staging model as a potential future standardised approach to improve clinical decision-making in real-world practice.

**Methods:**

A narrative synthesis of the Alzheimer’s disease literature was conducted to evaluate the relationship between ATN classification and clinical outcomes. Peer-reviewed studies were identified that examined ATN biomarkers across research and clinical settings, including longitudinal cohort studies, memory clinic populations and meta-analyses. Evidence was appraised to assess concordance and discordance between biological staging, cognitive severity, functional impairment and prognosis. Findings were synthesised thematically, focusing on clinical applicability, management implications and the potential role of an integrated clinical-biological staging model was evaluated.

**Results:**

Across many studies, the ATN framework demonstrated clear strengths in confirming Alzheimer’s disease pathology but consistent limitations in explaining clinical presentation. Amyloid positron emission tomography (PET) reliably confirmed underlying pathology but accumulated decades before symptom onset, therefore unable to predictcognitive severity. Tau PET and cerebrospinal fluid tau correlated closely with clinical symptoms but were limited by early sensitivity and spatial resolution. Neurodegeneration markers reflected synaptic dysfunction and brain atrophy but primarily represented late irreversible change. Overall, clinical symptoms and biomarker stage showed frequent discordance.

Evidence highlighted that discordant profiles were commonly influenced by resilience factors or additional co-pathologies not captured within the ATN framework. Integrated clinical-biological staging models map patients across two axes, which has been shown to better identify these profiles. Such models can more accurately reflect real-world heterogeneity.

**Conclusion:**

This evidence-based analysis demonstrates that while ATN-based diagnosis provides a strong biological framework, it is insufficient when used in isolation to explain symptom severity, prognosis or clinical variability. Integrated clinical-biological staging offers a more meaningful approach with implications for more accurate prognosis and targeted management. However, the clinical utility of this approach is currently limited by restricted availability of co-pathology biomarkers and the need for validation of integrated models in diverse populations. Therefore, further research is required before widespread standardised implementation.

